# Perception of the COVID-19 pandemic by individuals who previously sought psychiatric assistance

**DOI:** 10.1192/j.eurpsy.2022.657

**Published:** 2022-09-01

**Authors:** O. Boyko, T. Medvedeva, S. Enikolopov, O. Vorontsova, O. Kazmina

**Affiliations:** Federal State Budgetary Scientific institution “Mental health research center”, Clinical Psychology, Moscow, Russian Federation

**Keywords:** perception of the pandemic, Anxiety, Covid-19, Depression

## Abstract

**Introduction:**

COVID-19 pandemic leads to high levels of stress. Individuals who have previously sought psychiatric assistance are more sensitive.

**Objectives:**

Analysis of the perception of the pandemic by people who have previously sought psychiatric care.

**Methods:**

An internet-survey (20.03.2020 - 13.01.2021) (N=659; 152 – previously sought psychiatric assistance); included SCL-90-R; questions about the levels of anxiety, depression, and fear (assessed on 0-10 scale); question about opinion on COVID-19 pandemic (coded further on the basis of meaning); question about epidemiological situation of COVID-19 in respondents’ places of residence and their social circles.

**Results:**

Individuals who had previously sought psychiatric assistance demonstrated higher levels of anxiety (5,533±2,489 versus 4,774±2,590), depression (4,945±2,926 versus 3,861±2,988), and fear (0,195±0,397 versus 0,278±0,448). They showed roughly equivalent reactions to both anticipated and real danger (z-score GSI of SCL-90-R 0,90 versus 0,90 for anticipated and real danger respectively), the same indicator of the control group (0,53 and 0,65). In statements about the pandemic, they are more often referred to the topic of “positive effects” of pandemic (3,30% versus 0,99%), expressed “curiosity” (5,92% versus 2,37%). They were less drawn to conspiracy (9,87% versus 16,17%), and exploited more readily the topic “about myself” (20,39% versus 13,21%), negative images of “the present” (3,64% versus с 1,58%) and “the future” (15,79% versus 9, 47%), vocabulary of “anger” (5,92% versus 2,17%).

**Conclusions:**

Individuals who had previously sought psychiatric assistance were ambivalent in their attitudes towards pandemic, and tended to concentrate more on feelings and the negative vision of the future. They perceived anticipated danger roughly equivalent to real danger.

**Disclosure:**

No significant relationships.

